# Effectiveness of doctors’ advice on non-prescription antibiotic use: a randomized controlled trial, China

**DOI:** 10.2471/BLT.25.293840

**Published:** 2025-11-12

**Authors:** Minzhi Xu, Jianxiong Wu, Tenghao Wang, Chuangliang Qiu, Yuxin Zhao, Hui Li, Qihua Song, Yanhong Gong, Zuxun Lu, Xiaolin Wei, Xiaoxv Yin

**Affiliations:** a Department of Social Medicine and Health Management, Huazhong University of Science and Technology, Hangkong Road, Wuhan, 430030, China.; b Department of Public Health, the First Affiliated Hospital of Zhejiang Chinese Medical University, Hangzhou, China.; c Management of Community Health Service Center, Fuyong People’s Hospital of Baoan District, Shenzhen, China.; d School of Health Services Management, Anhui Medical University, Hefei, China.; e Dalla Lana School of Public Health, University of Toronto, Toronto, Canada.

## Abstract

**Objective:**

To evaluate a family doctor-led, community-based intervention to reduce non-prescription antibiotic use.

**Methods:**

We conducted a parallel-group, cluster-randomized controlled trial at 22 community health centres in Shenzhen, China, over an 8-month period in 2023. We randomly (1 : 1) assigned community health centres to provide a 4-week, family doctor-led, community-based online health intervention, or to provide routine care only. Eligible participants were adults aged 18 to 75 years who had resided in the community for more than 6 months. The primary outcome was the level of non-prescription antibiotic use (including self-medication with antibiotics and purchase of antibiotics without a prescription). Secondary outcomes were: levels of self-medication with antibiotics; purchase of antibiotics without a prescription; self-storage of antibiotics; and prescribed antibiotic use.

**Findings:**

We enrolled 1550 participants, with 788 assigned to the intervention group and 762 to the control group. We observed a significant decrease in non-prescription antibiotic use in the intervention group compared to the control group (odds ratio, OR: 0.49; 95% confidence interval, CI: 0.31–0.77) at 6 months. There was a significant reduction in self-medication (OR: 0.33; 95% CI: 0.13–0.83) and purchase of antibiotics without a prescription (OR: 0.59; 95% CI: 0.37–0.94), but not in self-storage (OR: 0.80; 95% CI: 0.54–1.18) or prescribed antibiotic use (OR: 0.94; 95% CI: 0.48–1.87) at 6 months.

**Conclusion:**

The family doctor-led, community-based intervention demonstrated promising effectiveness and feasibility. This study provides valuable insights for the design and implementation of such interventions aimed at promoting rational use of antibiotics.

## Résumé

**Objectif:**

Évaluer une intervention communautaire menée par des médecins de famille visant à réduire l’utilisation d’antibiotiques sans ordonnance.

**Méthodes:**

Nous avons mené un essai contrôlé randomisé en grappes avec groupes parallèles dans 22 centres de santé communautaires à Shenzhen, en Chine, sur une période de 8 mois en 2023. Nous avons attribué, de manière aléatoire (selon un rapport 1:1), à des centres de santé communautaires la mission de proposer une intervention sanitaire communautaire en ligne sur 4 semaines, menée par des médecins de famille, ou bien de fournir uniquement des soins de routine. Les participants éligibles étaient des adultes âgés de 18 à 75 ans résidant dans la communauté depuis plus de 6 mois. Le critère de jugement principal était le niveau de consommation d’antibiotiques sans ordonnance (y compris l’automédication à l’aide d’antibiotiques et l’achat d’antibiotiques sans ordonnance). Les critères de jugement secondaires étaient les suivants: niveaux d’automédication à l’aide d’antibiotiques; achat d’antibiotiques sans ordonnance; conservation d’antibiotiques à domicile; et utilisation d’antibiotiques sur ordonnance.

**Résultats:**

Nous avons recruté 1 550 participants, dont 788 ont été affectés au groupe d’intervention et 762 au groupe témoin. Nous avons observé une diminution significative de la consommation d’antibiotiques sans ordonnance dans le groupe d’intervention par rapport au groupe témoin (odds ratio (OR): 0,49; intervalle de confiance (IC) à 95%: 0,31–0,77) à 6 mois. Une réduction significative de l’automédication (OR: 0,33; IC à 95%: 0,13–0,83) et de l’achat d’antibiotiques sans ordonnance (OR: 0,59; IC à 95%: 0,37–0,94) a été observée, mais pas de la conservation d’antibiotiques à domicile (OR: 0,80; IC à 95%: 0,54–1,18) ni dans la consommation d’antibiotiques sur ordonnance (OR: 0,94; IC à 95%: 0,48–1,87) à 6 mois.

**Conclusion:**

L’intervention communautaire menée par des médecins de famille s’est révélée prometteuse en termes d’efficacité et de faisabilité. Cette étude fournit des renseignements précieux pour le design et la mise en œuvre de telles interventions visant à promouvoir l’utilisation rationnelle d’antibiotiques.

## Resumen

**Objetivo:**

Evaluar una intervención comunitaria dirigida por médicos de familia para reducir el uso de antibióticos sin prescripción médica.

**Métodos:**

Se realizó un ensayo clínico aleatorizado por conglomerados, de grupos paralelos, en 22 centros de salud comunitarios de Shenzhen (China) durante un periodo de ocho meses en 2023. Los centros se asignaron aleatoriamente (1:1) a ofrecer una intervención sanitaria en línea de cuatro semanas, basada en la comunidad y dirigida por médicos de familia, o a proporcionar únicamente la atención habitual. Los participantes elegibles fueron adultos de entre 18 y 75 años que hubieran residido en la comunidad durante más de seis meses. El desenlace principal fue el nivel de uso de antibióticos sin prescripción médica (incluido el autotratamiento con antibióticos y la compra de antibióticos sin prescripción). Los desenlaces secundarios fueron los niveles de autotratamiento con antibióticos, compra de antibióticos sin prescripción, almacenamiento doméstico de antibióticos y uso de antibióticos prescritos.

**Resultados:**

Se inscribieron 1550 participantes: 788 asignados al grupo de intervención y 762 al grupo de control. A los seis meses se observó una disminución significativa del uso de antibióticos sin prescripción médica en el grupo de intervención en comparación con el grupo de control (razón de posibilidades, OR: 0,49; intervalo de confianza del 95%, IC: 0,31-0,77). También se registró una reducción significativa del autotratamiento (OR: 0,33; IC del 95%: 0,13-0,83) y de la compra de antibióticos sin prescripción médica (OR: 0,59; IC del 95%: 0,37-0,94), aunque no se observaron cambios significativos en el almacenamiento doméstico (OR: 0,80; IC del 95%: 0,54-1,18) ni en el uso de antibióticos prescritos (OR: 0,94; IC del 95%: 0,48-1,87).

**Conclusión:**

La intervención comunitaria dirigida por médicos de familia demostró una eficacia y viabilidad prometedoras. Este estudio aporta información valiosa para el diseño y la aplicación de intervenciones orientadas a promover el uso racional de los antibióticos.

## ملخص

**الغرض:**

تقييم تدخل مجتمعي بقيادة طبيب الأسرة للحد من استخدام المضادات الحيوية دون وصفة طبية.

**الطريقة:**

قمنا بإجراء تجربة عنقودية عشوائية تخضع للتحكم، ومتوازية المجموعات، في 22 مركزًا صحيًا مجتمعيًا في شينزين بالصين، على مدار 8 أشهر في عام 2023. قمنا عشوائيًا بتخصيص (1:1) مراكز صحية مجتمعية لتقديم تدخل صحي مجتمعي عبر الإنترنت بقيادة طبيب للأسرة لمدة 4 أسابيع، أو لتقديم الرعاية الروتينية فقط. كان المشاركون المؤهلون بالغين تتراوح أعمارهم بين 18 و75 عامًا، ممن أقاموا في المجتمع لأكثر من 6 أشهر. تمثلت النتيجة الرئيسية في مستوى استخدام المضادات الحيوية دون وصفة طبية (بما في ذلك الطب الذاتي باستخدام المضادات الحيوية وشراء المضادات الحيوية دون وصفة طبية). أما النتائج الثانوية فكانت: مستويات الطب الذاتي باستخدام المضادات الحيوية؛ وشراء المضادات الحيوية دون وصفة طبية؛ والتخزين الذاتي للمضادات الحيوية؛ واستخدام المضادات الحيوية الموصوفة.

**النتائج:**

قمنا بتسجيل 1550 مشاركًا، منهم 788 في مجموعة التدخل و762 في مجموعة التحكم. لاحظنا انخفاضًا ملموسًا في استخدام المضادات الحيوية دون وصفة طبية في مجموعة التدخل مقارنةً بمجموعة التحكم (نسبة الأرجحية، فاصل ثقة: 0.49؛ بفاصل ثقة مقداره %95: 0.31 إلى 0.77) في 6 أشهر. كان هناك انخفاض ملموس في الطب الذاتي (نسبة الأرجحية: 0.33؛ ‏بفاصل ثقة مقداره %95: 0.13 إلى 0.83) وشراء المضادات الحيوية دون وصفة طبية (نسبة الأرجحية: 0.59؛ ‏بفاصل ثقة مقداره %95: 0.37 إلى 0.49)، ولكن ليس في التخزين الذاتي (نسبة الأرجحية: 0.80؛ ‏بفاصل ثقة مقداره %95: 0.54 إلى 1.18) أو استخدام المضادات الحيوية بوصفة طبية (نسبة الأرجحية: 0.94؛ ‏بفاصل ثقة مقداره %95: 0.48 إلى 1.87) في 6 أشهر.

**الاستنتاج:**

أظهر التدخل المجتمعي بقيادة طبيب الأسرة فعاليةً وإمكانيةً واعدتين. تُقدم هذه الدراسة رؤىً ذات قيمة لتصميم وتنفيذ مثل هذه التدخلات التي تهدف إلى تعزيز الاستخدام المتعقل للمضادات الحيوية.

## 摘要

**目的:**

旨在评估由家庭医生主导的基于社区的干预措施的有效性，从而减少非处方抗生素的使用。

**方法:**

我们于 2023 年在中国深圳的 22 个社区卫生中心开展了一项为期 8 个月的平行组整群随机对照试验。我们将这些社区卫生中心随机（按照 1:1 的比例）分配至两个试验组，为其中一组提供为期 4 周的由家庭医生主导的基于社区的在线卫生干预措施，为另一组则仅提供常规护理。将参与者的招募要求设定为年龄介于 18 至 75 岁之间且已在该社区居住 6 个月以上的成年人。主要结局为非处方抗生素使用程度（包括使用抗生素自行用药的情况和不凭医生处方购买抗生素的情况）。次要结局为：使用抗生素自行用药的程度；不凭医生处方购买抗生素的情况；自备抗生素的情况；以及处方抗生素使用的情况。

**结果:**

我们共招募了 1,550 名参与者，其中 788 名被分配至试验组，其余 762 名则被分配至对照组。我们发现，在第 6 个月时，与对照组相比，试验组的非处方抗生素使用【比值比 (OR)：0.49；95% 置信区间 (CI)：0.31-0.77】情况明显减少。在第 6 个月时，自行用药（OR：0.33；95% CI：0.13-0.83）和不凭医生处方购买抗生素（OR：0.59；95% CI：0.37-0.94）的情况明显减少，但是，自备抗生素（OR：0.80；95% CI：0.54-1.18）或处方抗生素使用（OR：0.94；95% CI：0.48-1.87）的情况并未明显减少。

**结论:**

研究结果表明，由家庭医生主导的基于社区的干预措施的有效性和可行性颇为显著。本项研究为旨在推广合理使用抗生素的此类干预措施的制订和实施提供了宝贵见解。

## Резюме

**Цель:**

Оценить влияние вмешательства, проводимого под руководством семейного врача, в общине с целью сокращения безрецептурного использования антибиотиков.

**Методы:**

На протяжении 8 месяцев 2023 года авторы проводили контролируемое кластерно-рандомизированное исследование в параллельных группах в 22 общественных центрах здравоохранения в Шеньчжене (Китай). Общественные центры здравоохранения были случайным образом (1:1) распределены для проведения онлайнового вмешательства, осуществляемого в общине семейным врачом на протяжении 4 недель, или только для планового медицинского обслуживания. К участию в исследовании допускались взрослые в возрасте от 18 до 75 лет, которые прожили в общине не менее 6 месяцев. Основным результатом исследования был уровень безрецептурного использования антибиотиков (включая самолечение антибиотиками и приобретение антибиотиков без рецепта). В качестве вторичных результатов исследования рассматривались: уровни самолечения антибиотиками, приобретение антибиотиков без рецепта, самостоятельное хранение антибиотиков и использование антибиотиков по рецепту.

**Результаты:**

В исследование было включено 1550 человек, из них 788 человек попали в группу вмешательства и 762 человека попали в контрольную группу. Через 6 месяцев наблюдалось значительное сокращение случаев безрецептурного использования антибиотиков в группе вмешательства по сравнению с контрольной группой (отношение шансов, ОШ: 0,49; 95%-й доверительный интервал, ДИ: 0,31–0,77). Через 6 месяцев также значительно снизились показатели самолечения (ОШ: 0,33; 95%-й ДИ: 0,13–0,83) и приобретения антибиотиков без рецепта (ОШ: 0,59; 95%-й ДИ: 0,37–0,94), однако это не относилось к самостоятельному хранению антибиотиков (ОШ: 0,80; 95%-й ДИ: 0,54–1,18) или использованию антибиотиков по рецепту (ОШ: 0,94; 95%-й ДИ: 0,48–1,87).

**Вывод:**

Вмешательство под руководством семейного врача на уровне общины продемонстрировало осуществимость и многообещающую эффективность. Данное исследование предоставляет ценную информацию относительно планирования и реализации таких вмешательств, направленных на поощрение рационального использования антибиотиков.

## Introduction

Antimicrobial resistance has emerged as a critical global public health challenge.^1^^,^^2^ As well as the overprescription of antibiotics in clinical settings, non-prescription use by the public is also a concern.^3^^–^^5^ A wide variety of antibiotics and their complex pharmacological mechanisms makes it difficult for the public to use antibiotics appropriately without guidance from medical professionals.^6^^,^^7^ Non-prescription antibiotic use can also delay individual diagnosis and treatment and increase the risk of developing and spreading multidrug-resistant organisms within the community.^8^

Implementing interventions to reduce non-prescription antibiotic use presents numerous challenges. A critical factor contributing to ineffective interventions is the knowledge–do gap, with evidence showing that there is no linear relationship between increased knowledge and improved antibiotic use among the public.^9^^,^^10^ Therefore, knowledge campaigns alone may not effectively curb irrational antibiotic use. Behavioural science suggests that changing behaviour requires sustained professional involvement and reinforcement.^11^^,^^12^ Family doctors play a pivotal role in public health education due to established long-term relationships and trusted positions within the community.^13^ Regular health education conducted by family doctors offers a sustainable approach to disseminating health knowledge, correcting misconceptions and addressing irrational antibiotic use.^14^^,^^15^ The family doctor system is also integrated within the community health service network and can leverage multisectoral collaboration to deliver large-scale public health interventions.^16^^,^^17^

Given the lack of established intervention models for public antibiotic use, we developed a family doctor-led, community-based intervention aimed at reducing non-prescription antibiotic use. We then tested its effectiveness through a cluster-randomized controlled trial, which we report here.

## Methods

### Study design and setting

We conducted a parallel-group, cluster-randomized controlled trial in 22 communities in the Baoan District of Shenzhen, in Guangdong Province, China, in 2023. In this trial, we compared a family doctor-led, community-based intervention with routine care to assess the intervention’s effectiveness in reducing non-prescription antibiotic use. We collected data over an 8-month period.

Baoan is a rapidly urbanizing area with a permanent resident population exceeding 4 million. To meet primary health care demands, Baoan has established a network of over 180 community health centres, forming a primary care system centred on the family doctor contract service. Family doctors in China typically receive shorter specialized training compared to their counterparts in high-income countries. Additionally, their scope of practice is more focused on public health and chronic disease monitoring rather than comprehensive primary care. The community health centres involved in this study are affiliated with Fuyong People’s Hospital and serve diverse communities, including urban built-up areas, urban-rural transitional zones and industrial clusters. Each community health centre is staffed with general practitioners, nurses and public health personnel delivering integrated services such as basic medical care, health education and chronic disease management. The general practitioners in our study were all certified family doctors.

We conducted an internal pilot process before initiating the main trial: we enrolled four community health centres as pilot sites and randomized them to either the intervention or control arm. We followed all sites up for 1 month to assess the feasibility and acceptability of the intervention. After confirming the intervention’s feasibility and acceptability, we extended it to the remaining health centres. No changes needed to be made to the study implementation processes between the pilot and main intervention stages.

### Eligibility

Our inclusion criteria for community health centres were as follows: a resident population of at least 5000; the presence of fully registered family doctors at the community health centre; and all family doctors employed at the community health centre had at least 3 years of clinical experience in general practice. Hospital administration initially recommended all participating family doctors; doctors were subsequently reviewed and confirmed by the research team before inclusion.

Our inclusion criteria for participants were as follows: aged between 18 and 75 years; residence in the local community for more than 6 months; normal cognitive function, with voluntary, informed consent to participate; and ability to use the messaging service WeChat (Tencent, Shenzhen, China) independently or with assistance from a family member. Our exclusion criteria were: current employment in any community health centre (e.g. as a doctor, pharmacist, nurse or medical or pharmacy student); diagnosis of immune system disorders or hereditary conditions that may affect routine medication use; and pregnancy or breastfeeding. Community health centre family doctors recruited community members meeting the inclusion criteria.

### Randomization and blinding

After conducting a baseline survey among participants recruited from all 22 community health centres, we performed stratified randomization (1 : 1) to assign these centres to either the intervention or control group (routine care). The stratification was based on community population size (large: > 40 000 residents; medium: 20 000–40 000 residents; small: < 20 000 residents). We used a computer program written in R, version 4.0.5 (R Foundation for Statistical Computing, Vienna, Austria) for randomization. The nature of the intervention meant that blinding of family doctors and participants was not feasible. We therefore followed the PROBE design for this trial;^18^ we kept data analysts blinded to the group allocation.

### Intervention procedures

We developed a 4-week, family doctor-led, community-based online health intervention comprising four core components: establishment of WeChat health education groups; development of science-based educational articles; production of short educational videos; and implementation of telephone-based education. First, we established 22 WeChat groups, one for each participating community health centre. Eligible participants joined by scanning a WeChat QR (quick-response) code. The assigned family doctor managed each WeChat group; the doctor was responsible for disseminating educational materials and responding to participants’ inquiries regarding antibiotic use. Second, the research team identified key educational themes related to appropriate antibiotic use and developed four science-based articles. We selected 10 participants representing various educational levels to pilot test each article to ensure article readability. We incorporated feedback to refine language and improve clarity. Each article was designed to be read within 3 minutes, optimizing information conciseness and user engagement. Additionally, a multidisciplinary video production team, including researchers and family doctors, developed four short educational videos featuring family doctors as narrators. Each video lasted 2–3 minutes to maintain viewer attention. During the intervention period, family doctors posted one article and one corresponding video weekly via the official WeChat public account. After publication, the content was shared in the respective WeChat group with accompanying explanatory text. Video topics aligned with the weekly article theme to reinforce key messages. In the fourth week, family doctors conducted telephone-based education. Key components included: confirming whether participants had read or viewed the posted materials; assessing recent antibiotic use and emphasizing the risks of misuse; and reinforcing the importance of appropriate antibiotic use under medical supervision. To maximize call completion rates, we scheduled calls during one of three time windows: 11:00–12:00, 16:00–17:00, or 19:00–20:00. Each call lasted 5–10 minutes, balancing family doctors’ workload with participant acceptability.

Before the implementation of the intervention, all family doctors in the intervention group underwent an 8-hour training programme over two days, combining theoretical explanations with practical exercises. The theoretical component covered principles of appropriate antibiotic use, antimicrobial resistance and health communication strategies. The practical component included simulated interactions, such as responding to queries in the WeChat group, posting articles and videos, and conducting telephone-based education, to ensure that all family doctors could independently perform the intervention. We conducted scenario-based assessments post-training, with all family doctors successfully completing intervention steps.

### Control procedures

Participants attending community health centres assigned to the control arm received only routine public health education, which did not include any antibiotic-related content.

### Data collection

We collected baseline data before intervention implementation, with the first follow-up survey conducted 1 month after intervention and the second follow-up survey conducted 6 months after intervention. To ensure consistency and comparability across time points, we conducted both baseline and follow-up surveys using identical data collection methods. We collected data using a questionnaire that we had designed on the online survey platform “Questionnaire Star” (Changsha Ranxing Information Technology Co., Ltd, Changsha, China). The questionnaire had three parts: demographic information (including sex assigned at birth, age, education level, employment status, marital status, self-perceived health status and self-perceived economic status); non-prescription antibiotic use in the past month (including self-medication with antibiotics and purchase of antibiotics without a prescription; we collected additional data on self-storage of antibiotics and prescribed antibiotic use); process evaluation indicators for the intervention (including intervention satisfaction, acceptance, degree of comprehensibility of health education content, and appropriateness of intervention frequency). A designated reviewer monitored the survey platform daily to examine submitted questionnaires for completeness and data quality, providing feedback as needed.

### Outcomes

The primary outcome was the level of non-prescription antibiotic use (non-prescription antibiotic use being defined as either self-medication with antibiotics or purchase of antibiotics without a prescription). The primary outcome was assessed at three time points: baseline, the first follow-up (at 1 month) and the second follow-up (at 6 months). Secondary outcomes were the level of two specific non-prescription antibiotic use behaviours: self-medication with antibiotics and purchase of antibiotics without a prescription, to disentangle the distinct components of non-prescription use. We measured behaviours using the following two questions: (i) “In the last month, have you self-medicated with antibiotics without a prescription?” and (ii) “In the last month, have you purchased antibiotics without a prescription?” Each question assessed a specific type of non-prescription antibiotic use behaviour, with dichotomous (“Yes” or “No”) responses recorded. We also included the level of self-storage of antibiotics as a secondary outcome as this may reflect antibiotic availability and preparedness. Additionally we measured prescribed antibiotic use; this measure allowed us to distinguish non-prescription behaviours from regulated use and to assess whether reductions in non-prescription antibiotic use were accompanied by a corresponding increase in doctor-directed (prescription) antibiotic use, potentially indicating a substitution effect.

We conducted a process evaluation 1 month after completing the intervention. We measured participants’ satisfaction, acceptance and intervention comprehensibility using five-point Likert scales. Frequency appropriateness was categorized as appropriate, excessive or insufficient.

### Statistical analysis

We estimated the number of community centres that would be required for each arm of our study as follows: based on a cross-sectional survey of non-prescription antibiotic use conducted in the Baoan District before the start of our trial, we estimated that the prevalence of non-prescription antibiotic use in this area was 18% (261/1488). We hypothesized that the intervention would reduce the non-prescription antibiotic rate by 40%. To detect this effect with 80% power at a two-sided 5% significance level, assuming an average cluster size of 50 participants, an intracluster correlation coefficient of 0.01 and accounting for an estimated 20% loss to follow-up, we determined that 11 community health centres were required per study arm, with 660 participants per arm.^19^ We therefore included a total of 22 community health centres in our study.

We performed all analyses in the intention-to-treat population. We reported per-protocol analyses to assess the robustness of our results. We used separate mixed-effects logistic regression models to assess the intervention effect on each outcome. All models included fixed effects for time, group, and the time-by-group interaction, adjusted for baseline values, gender, age, employment status, marital status, self-perceived health status and self-perceived economic status. We included community health centres as a random intercept to account for correlation among participants within the same centre. The time-by-group interaction coefficient was the primary estimate of the intervention effect. We identified no missing data for the covariates. We conducted all analyses using complete cases and used R (version 4.0.5) for all statistical analyses. Two-sided *P* < 0.05 was considered statistically significant for all analyses.

This trial is registered with ClinicalTrials.gov, NCT06317415.

### Ethics review

The trial was reviewed and approved by the Medical Ethics Committee of Fuyong People’s Hospital, with approval number KY-2022–5. All participants provided written informed consent before enrolment.

## Results

**Fig. 1:**
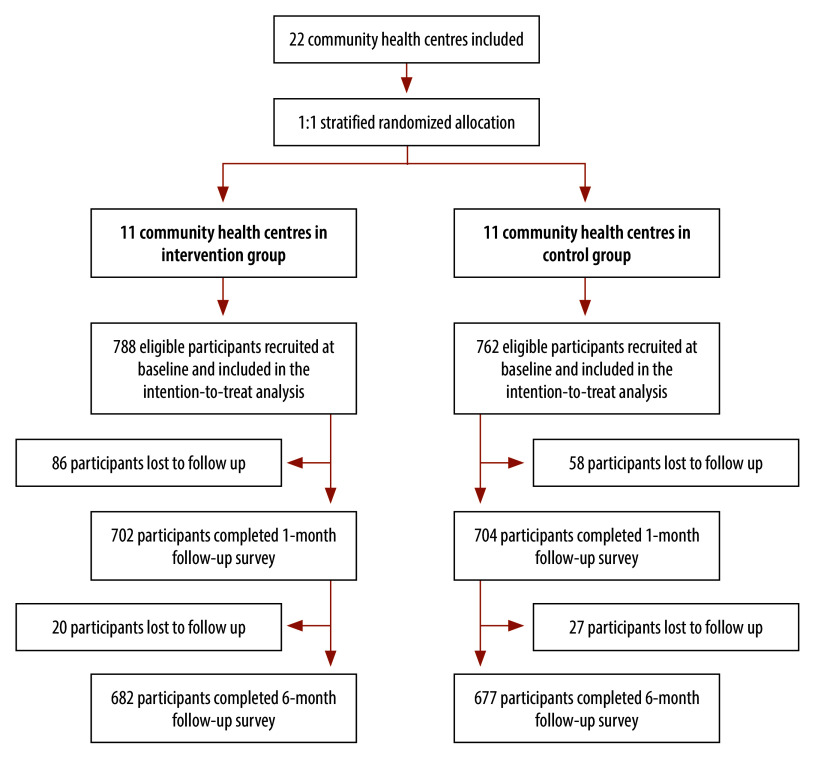
Flowchart showing cluster-randomized controlled trial profile, China, 2023

The trial was conducted from 1 April 2023 to 31 December 2023. During the baseline phase, we enrolled a total of 1550 eligible participants, with 788 assigned to the intervention group and 762 to the control group (Fig. 1). At 6-month follow-up, 191 participants (106 in the intervention group and 85 in the control group) were lost to follow-up, yielding an overall attrition rate of 12% (191/1550). There was no significant difference in the loss to follow-up proportions between the intervention and control groups (online repository).^20^ Finally, 1359 participants completed the follow-up phases, with 682 in the intervention group and 677 in the control group. Overall, the baseline sociodemographic characteristics of the study participants were well balanced between the groups, with some modest (> 5%) imbalances in participants’ employment status (Table 1). The median duration of family doctor contracting experience was 6.0 years (interquartile range, IQR: 4.5–7.5) in the intervention group, slightly lower than that in the control group (7.0 years; IQR: 4.0–10.0). The intervention arm involved a smaller proportion of male family doctors (2/11; 18%) compared to the control group (4/11; 36%). The distribution of doctors' educational levels was identical between the two groups (online repository).^20^

**Table 1:** Baseline sociodemographic characteristics of participants in a cluster-randomized controlled trial of doctors’ advice on non-prescription antibiotic use, China, 2023

Sociodemographic characteristic	No. (%)		
	Both arms (*n* = 1550)	Intervention arm (*n* = 788)	Control arm (*n* = 762)
**Sex**			
Male	639 (41.2)	337 (42.8)	302 (39.6)
Female	911 (58.8)	451 (57.2)	460 (60.4)
**Age (years)**			
≤ 25	196 (12.7)	95 (12.1)	101 (13.3)
26–44	963 (63.1)	498 (63.2)	465 (61.0)
45–59	321 (20.7)	164 (20.8)	157 (20.6)
≥ 60	70 (4.5)	31 (3.9)	39 (5.1)
**Education level**			
Junior high school and below	375 (24.2)	193 (24.5)	182 (23.9)
High school	415 (26.8)	216 (27.4)	199 (26.1)
Undergraduate and above	760 (49.0)	379 (48.1)	381 (50.0)
**Employment status**			
Full-time job	1032 (66.6)	553 (70.2)	479 (62.9)
Part-time job	120 (7.7)	66 (8.4)	54 (7.1)
Unemployed	231 (14.9)	100 (12.7)	131 (17.2)
Other	167 (10.8)	69 (8.8)	98 (12.9)
**Marital status**			
Unmarried	337 (21.7)	170 (21.6)	167 (21.9)
Married	1177 (75.9)	600 (76.1)	577 (5.7)
Divorced or widowed	36 (2.3)	18 (2.3)	18 (2.4)
**Self-perceived health status**			
Poor	123 (7.9)	66 (8.4)	57 (7.5)
Average	800 (51.6)	417 (52.9)	383 (50.3)
Good	627 (40.5)	305 (38.7)	322 (42.3)
**Self-perceived economic status**			
Poor	212 (13.7)	109 (13.8)	103 (13.5)
Average	1190 (76.8)	594 (75.4)	596 (78.2)
Good	148 (9.5)	85 (10.8)	63 (8.3)

Note: inconsistencies arise in some values due to rounding.

**Fig. 2:**
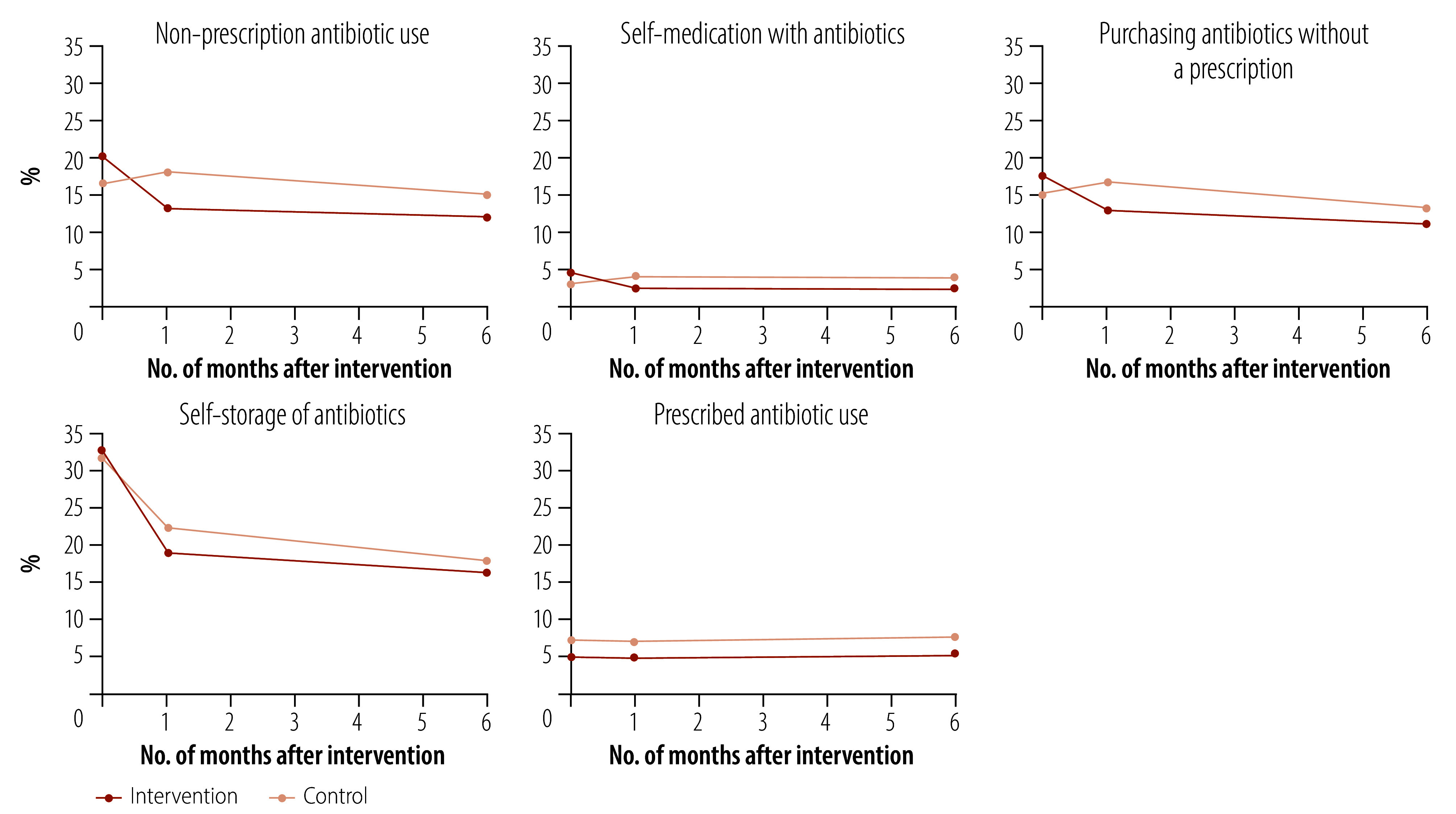
Changes in non-prescription antibiotic use, cluster-randomized controlled trial, China, 2023

Note: Absolute numbers used to calculate the percentages are available in Table 2.

Levels of non-prescription antibiotic use, self-medication with antibiotics, purchase of antibiotics without a prescription, self-storage of antibiotics, and use of prescription antibiotics in the intervention and control groups over time are presented in Fig. 2 and Table 2 (available at: https://www.who.int/publications/journals/bulletin). There was a significant decrease in non-prescription antibiotic use in the intervention group compared to the control group at 1 month after the intervention (odds ratio, OR:0.45; 95% confidence interval, CI: 0.29–0.69) and at 6 months (OR: 0.49; 95% CI: 0.31–0.77). In terms of secondary outcomes, there was a significant decrease in self-medication with antibiotics (OR: 0.36; 95% CI: 0.15–0.89 at 1 month and OR: 0.33; 95% CI: 0.13–0.83 at 6 months) and the purchase of antibiotics without a prescription (OR: 0.54; 95% CI: 0.35–0.84 at 1 month and OR: 0.59; 95% CI: 0.37–0.94 at 6 months) in the intervention group compared to the control group. However, the intervention had no significant effect on self-storage of antibiotics (OR: 0.73; 95% CI: 0.50–1.07 at 1 month and OR: 0.80; 95% CI: 0.54–1.18 at 6 months) and prescribed antibiotic use (OR: 0.91; 95% CI: 0.45–1.82 at 1 month and OR: 0.94; 95% CI: 0.48–1.87 at 6 months). The results of the per-protocol analyses were consistent with those of the intention-to-treat analyses (Table 3).

**Table 2:** Changes in non-prescription antibiotic use, cluster-randomized controlled trial, China, 2023

Behaviour	No./n		
	Baseline	One month after intervention	Six months after intervention
**Non-prescription antibiotic use**			
Intervention	159/788	94/702	83/682
Control	126/762	127/704	102/677
**Self-medication with antibiotics**			
Intervention	35/788	18/702	16/682
Control	23/762	28/704	26/677
**Purchasing antibiotics without a prescription**			
Intervention	138/788	90/702	75/682
Control	114/762	116/704	88/677
**Self-storage of antibiotics**			
Intervention	258/788	134/702	112/682
Control	241/762	157/704	122/677
**Prescription antibiotic use**			
Intervention	39/788	32/702	36/682
Control	53/762	48/704	50/677

**Table 3:** Effect of doctors’ advice on non-prescription antibiotic use, China, 2023

Behaviour	Intention to treat OR^a^ (95% CI)	Per protocol OR^a^ (95% CI)
**Primary outcome**		
Non-prescription antibiotic use		
Baseline	1.00	1.00
1 month after intervention	0.45 (0.29–0.69)	0.46 (0.29–0.71)
6 months after intervention	0.49 (0.31–0.77)	0.50 (0.32–0.79)
**Secondary outcomes**		
Self-medication with antibiotics		
Baseline	1.00	1.00
1 month after intervention	0.36 (0.15–0.89)	0.40 (0.16–1.00)
6 months after intervention	0.33 (0.13–0.83)	0.35 (0.14–0.88)
Purchase of antibiotics without a prescription		
Baseline	1.00	1.00
1 month after intervention	0.54 (0.35–0.84)	0.53 (0.34–0.84)
6 months after intervention	0.59 (0.37–0.94)	0.57 (0.36–0.92)
Self-storage of antibiotics		
Baseline	1.00	1.00
1 month after intervention	0.73 (0.50–1.07)	0.76 (0.52–1.11)
6 months after intervention	0.80 (0.54–1.18)	0.79 (0.53–1.18)
Prescription antibiotic use		
Baseline	1.00	1.00
1 month after intervention	0.91 (0.45–1.82)	0.95 (0.47–1.95)
6 months after intervention	0.94 (0.48–1.87)	0.90 (0.45–1.80)

^a^ Adjustment variables were baseline values, sex, age, employment status, marital status, self-perceived health status and self-perceived economic status.

**Fig. 3:**
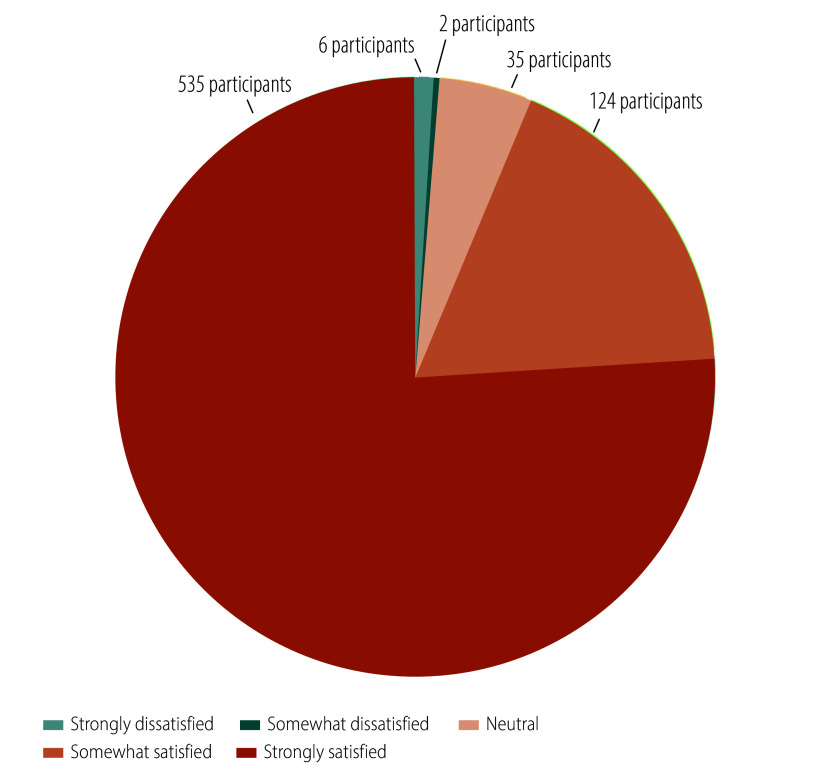
Evaluation of the intervention satisfaction among the study population, cluster-randomized controlled trial, China, 2023

Note: 702 participants completed the online survey 1 month after the intervention.

**Fig. 4:**
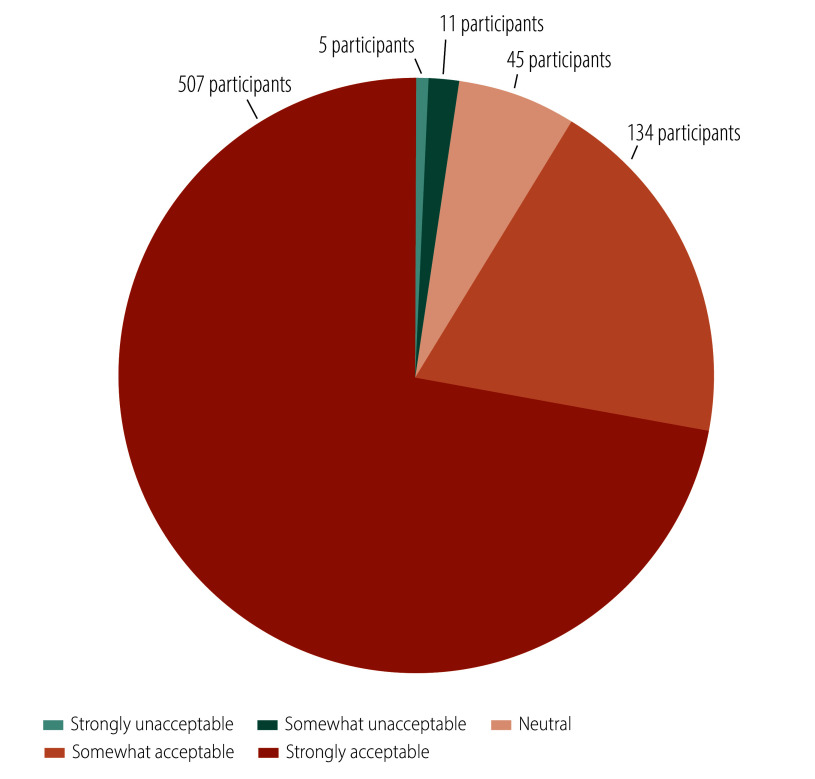
Evaluation of the intervention acceptance among the study population, cluster-randomized controlled trial, China, 2023

Note: 702 participants completed the online survey 1 month after the intervention.

**Fig. 5:**
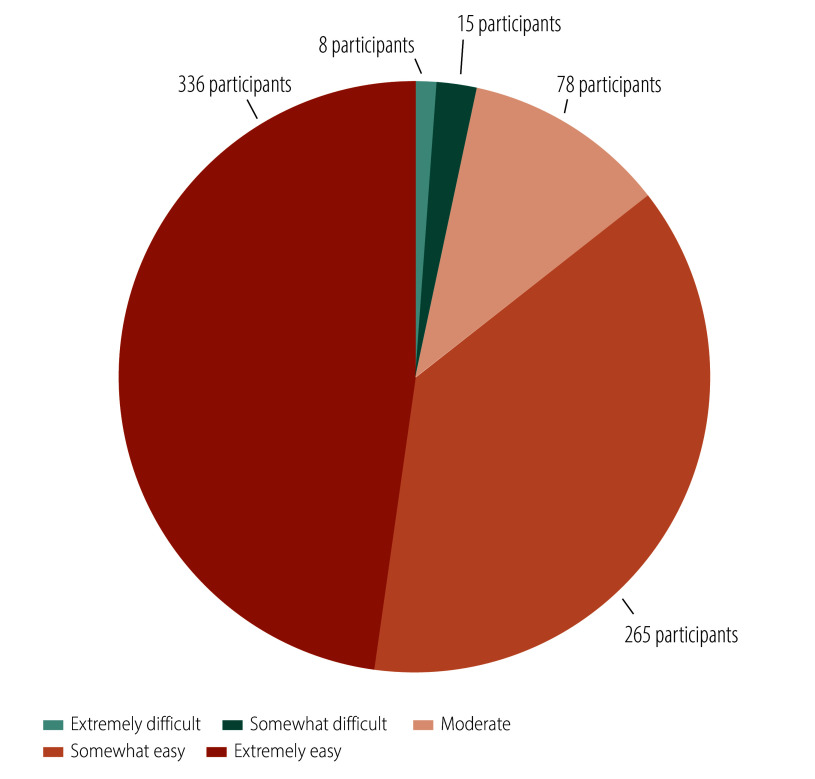
Evaluation of the degree of comprehensibility of health education content among the study population, cluster-randomized controlled trial, China, 2023

Note: 702 participants completed the online survey 1 month after the intervention.

**Fig. 6:**
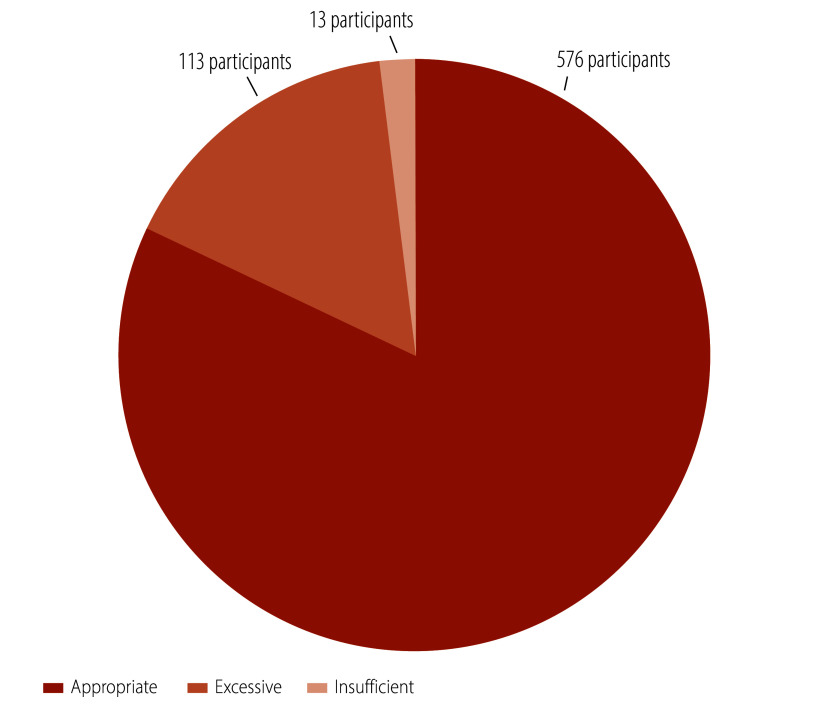
Evaluation of the appropriateness of intervention frequency among the study population, cluster-randomized controlled trial, China, 2023

Note: 702 participants completed the online survey 1 month after the intervention.

We conducted a process evaluation one month after the intervention. Regarding satisfaction, 76% (535/702) of participants reported being strongly satisfied with the intervention, while 18% (124/702) were somewhat satisfied (Fig. 3). In terms of acceptance, 72% (507/702) found the online health education highly acceptable, and 19% (134/702) found it somewhat acceptable (Fig. 4). Concerning the comprehensibility of the health education content, 48% (336/702) found it extremely easy to understand while 38% (265/702) found it somewhat easy (Fig. 5). Regarding the appropriateness of the intervention frequency, 82% (576/702) of participants considered it appropriate (Fig. 6).

## Discussion

Our cluster-randomized controlled trial demonstrates that a family doctor-led, online health intervention significantly reduced non-prescription antibiotic use among community residents. Both components of non-prescription use – self-medication with antibiotics and purchase of antibiotics without a prescription – showed meaningful improvements. The observed reduction in self-medication and non-prescription purchasing aligns with the intervention’s core components, which emphasized education on antibiotic indications, risks of misuse and the importance of professional consultation.

Notably, levels of antibiotic self-storage and prescribed antibiotic use showed no significant differences between the intervention and control groups over time. The reduction in self-storage of antibiotics coincided with a modest increase in prescribed antibiotic use, a pattern likely driven by broader epidemiological and seasonal fluctuations in respiratory infections, rather than the intervention itself. This trial was conducted in the first year after the Chinese government had relaxed its strict coronavirus disease 2019 control policies, a period characterized by temporarily increased susceptibility to infections, due to reduced pathogen exposure during the pandemic. As population immunity waned, there was a widespread resurgence of respiratory infectious diseases, particularly during the autumn and winter months, leading to increased demand for antibiotic treatment.^21^ This surge may have prompted individuals to consume previously stockpiled antibiotics for self-treatment, explaining the substantial decline in self-storage of antibiotics observed in both groups.^22^ At the same time, heightened disease burden may have contributed to a slight rise in prescribed antibiotic use, consistent with seasonal patterns of respiratory infections.^23^^,^^24^ Importantly, this increase in prescribed antibiotic use with time did not exceed or negate the larger reduction in non-prescription use. While the intervention successfully reduced non-prescription antibiotic use, parallel changes in self-storage and prescription use highlight how external factors, such as post-pandemic infection dynamics, can at the same time shape antibiotic-related behaviours at the community level. Nevertheless, the fact that reduced self-storage was not accompanied by an excessive rise in antibiotic prescriptions indicates that our intervention is effective and can prompt a more rational use among the public.

Our process evaluation revealed high satisfaction and acceptance of the intervention, likely due in large part to the family doctor-led, community-based approach.^25^ Family doctors provided continuous follow-up on individual health, enhancing the continuity of antibiotic education. Despite good health-care access, non-prescription antibiotic use remains common because of over-the-counter availability and misconceptions about antibiotics.^26^ Family doctors offer evidence-based guidance that curbs inappropriate antibiotic use. Our study shows the value of integrating family doctors into community antibiotic stewardship.

Our trial has several limitations. First, we assessed antibiotic use through self-reported questionnaires, these may be subject to social desirability bias and recall inaccuracy.^27^ Although the survey was confidential and we used neutral questions to minimize these effects, we did not validate self-reports with objective data sources such as prescription databases, a common challenge in large-scale community-based studies.^28^^,^^29^ Second, the study took place in a post-pandemic period, when changing infection patterns and increased awareness of respiratory illnesses may have independently influenced antibiotic-related behaviours.^30^ These factors may limit generalizability of our findings to other populations or settings. Privacy constraints also prevented collection of individual diagnoses or test results to confirm the appropriateness of antibiotic use, so outcomes reflect rational use behaviour rather than clinical appropriateness.

Overall, our family doctor-led, community-based intervention showed promising effectiveness and feasibility. Following the intervention, non-prescription antibiotic use decreased significantly and remained at a low level half a year later, indicating a sustained behavioural impact. Our study provides valuable insights for family doctor-led interventions aimed at promoting the rational use of antibiotics.
